# V4020 Venezuelan Equine Encephalitis Vaccine: Mitigating Neuroinvasion and Reversion Through Rational Design

**DOI:** 10.3390/v17081136

**Published:** 2025-08-19

**Authors:** Adrian Centers, Koji Barnaby, Sidney Goedeker, Ava Pignataro, Irina Tretyakova, Igor Lukashevich, Peter Pushko, Donghoon Chung

**Affiliations:** 1 School of Medicine, University of Louisville, 505 S. Hancock Street, Louisville, KY 40202, USA; adrian.centers@louisville.edu (A.C.); koji.barnaby@louisville.edu (K.B.); sgoedeker@unr.edu (S.G.); avapignataro19@augustana.edu (A.P.); igor.lukashevich@louisville.edu (I.L.); 2 Medigen, Inc., 8420-S Gas House Pike, Frederick, MD 21701, USA; itretyakova@medigen-usa.com

**Keywords:** Venezuelan equine encephalitis, VEE, live attenuated VEEV vaccine, neuroinvasion, pseudoreversion

## Abstract

There is a need for safe and effective vaccines against the Venezuelan equine encephalitis virus that infects both humans and equines. However, development of a live-attenuated vaccine using the TC-83 strain has been hampered by substantial reactogenicity and the potential for neuroinvasion. In this study, we demonstrate that V4020, a new TC-83-based investigational VEEV vaccine with redundant safety features preventing neuroinvasion and reversion, exhibited no neuroinvasion potential in a murine model. Following subcutaneous or intramuscular administration, a subset of mice that received the TC-83 vaccine succumbed to central nervous system infection, with replicating virus detected in the CNS, demonstrating a low, yet detectable neuroinvasion potential of the TC-83 vaccine in vivo. Sequencing analysis of the TC-83 virus recovered from the brains identified a pseudoreversion of E2 R120I, as E2 R120 is known to confer attenuation for TC-83. In contrast, V4020 showed no evidence of virus in the CNS, highlighting one of the V4020 features, a new synonymous codon to minimize reversion to the wild-type residue. Overall, our study establishes V4020 as a rationally designed, safe vaccine candidate for VEEV with significantly reduced neuroinvasion risk.

## 1. Introduction

Venezuelan equine encephalitis (VEE) is caused by VEE virus (VEEV), an alphavirus transmitted by mosquitoes. VEEV has a positive-sense, single-stranded RNA genome of 11.5 kb in length [1]. The virion has an enveloped icosahedral structure approximately 70 nm in diameter. In humans, VEEV causes a biphasic febrile illness that can result in myeloencephalitis with high morbidity, including neurological complications and a mortality rate of approximately 2% [2]. The initial symptoms of VEE, such as fever and headache, resemble other infections, which complicates clinical diagnosis. In addition, there is considerable overlap between geographic regions of VEEV endemicity and those of some other tropical diseases. For example, an overlap of VEEV with areas of dengue virus may result in an underestimation of VEEV prevalence [3]. Ecological and climate factors may cause increases in the geographical distribution of Culex mosquitoes, transmitting VEEV to humans and equines at risk of contracting the virus [4]. VEEV is also recognized as a potential biological weapon, due to the severe threat posed to human and animal health, and the minimal infectious aerosol dose required for infection. Finally, VEEV has a history of laboratory-acquired infections [5,6,7]. Taken together, these factors suggest the continuing possibility of VEEV infections and outbreaks, including in non-endemic areas.

Currently, there is no approved VEEV vaccine or therapy for humans. The TC-83 vaccine, developed in the 1960s, has been used as an Investigational New Drug (IND) for vaccination of laboratory personnel at risk of infection with VEEV [8]. The vaccine induces neutralizing antibodies that protect from infection; however, there are several issues regarding the efficacy and safety of the TC-83 as a vaccine for humans. It has been reported that approximately 20% of recipients do not develop neutralizing titer, and more importantly, roughly 20% of vaccinees experience mild to moderate adverse reactions, ranging from inflammatory (e.g., fever, chills, and malaise) to neurological (e.g., anorexia) clinical reactions [8,9,10]. A formalin-inactivated VEEV vaccine, C-84, has been developed using TC-83 [11]. Although C-84 is found to be less reactogenic and less protective than TC-83, it provides an immune response in TC-83 non-responders and has been used as a booster with TC-83 [8].

Regarding the safety of VEEV vaccines, it is important to ensure the lack of neuroinvasive properties, especially for a live attenuated vaccine due to the neurotropic nature of its parental strain. Previous reports have demonstrated that attenuating mutations render the virus incapable of CNS invasion [12]. Immunocompetent mice are able to effectively control the attenuated VEEV strains after subcutaneous (SC) or intramuscular (IM) injections, preventing neuroinvasion. However, some studies have indicated that the VEEV live-attenuated vaccines may be able to gain access to the CNS from the peripheral inoculation site, resulting in a CNS infection in humans or in immunocompetent mice [12], particularly by a reversion of the attenuating mutations considering the high mutation rate of RNA viruses in general and high diversity of population within VEEV TC-83 sample [13].

To address this safety concern, we previously developed a live attenuated VEEV vaccine, V4020, which includes the key attenuating mutations of TC-83, namely 5′A3 and E2-Arg120 derived, as well as a genetic rearrangement of the capsid and glycoprotein genes for an additional attenuation factor [14,15,16] (Supplementary Figure S1). Importantly, to prevent potential reversions to the wild-type genotype, the translational codon of AGA for the attenuating mutation E2-Arg in the TC-83 was replaced with CGA, synonymous but requiring two point mutations to revert to the wild-type codon ACA encoding E2-Thr as in virulent VEEV strains [14,15]. These changes were designed to enhance the safety of the prospective vaccine for human use [17,18].

Here, we investigated the potential of neuroinvasion of V4020 in immunocompetent mice and found that V4020 has a considerably lower neuroinvasion potential than that of TC-83. While a few of the TC-83 vaccinated mice showed active viral replication in the CNS after a subcutaneous or intramuscular administration, none of the mice administered with V4020 showed any signs of virus replication in the brain. We found that the TC-83 virus isolated in the CNS has a mutation of R120I in the E2 gene, which might have served as a phenotypic reversion (i.e., pseudoreversion), providing a justification for our approach of using a new codon for the residue.

## 2. Materials and Methods

### 2.1. Vaccine Viruses

Test vaccine virus, V4020 (lot: 20230512), was generated as previously described [14,15] and titrated by plaque assay, with a titer of 9.33 × 10^7^ PFU/mL. The control vaccine stock, VEEV TC-83 (lot: 20230404/1), was amplified once in Vero cells with an MOI of 0.001 from the TC-83 vaccine (USAMRIID, Fort Detrick, MD, USA) and used as a comparator, with a titer of 1.11 × 10^9^ PFU/mL. The stock viruses were stored at −80 °C until used. The seed stock of each vaccine virus was diluted to the desired concentration in phosphate-buffered saline (PBS) and kept on ice before injections.

### 2.2. Neuroinvasion Animal Model

Mice, BALB/c (Jackson Laboratory, Bar Harbor, ME, USA, Cat. # 000651), 4–6 weeks old, were used for SC and IM administration studies. Animals were acclimated 3–5 days prior to being used for experiments. Toe tattoos were applied for identification of individual animals, and three or four same sex mice were housed together as a group. For administration, 2 × 10^6^ pfu of virus was administered via either SC injection (50 µL per injection) between the shoulder blades or IM injection of 25 µL of dilute virus into each hind leg (total volume of 50 µL per mouse). For the mock infected group, PBS was used in place of the virus. For the intranasal (IN) infection model, mouse strain C3H (Charles River Laboratory, Wilmington, MA, USA, Cat. # 025), 4–6 weeks old, was used [19]. After an acclimatization period, diluted vaccine was administered via the IN route in a total volume of 50 µL (25 µL/naris) under anesthesia with isoflurane. All procedures in mice were carried out in accordance with the American Association for Accreditation of Laboratory Animal Care and institutional guidelines for animal welfare and use, reviewed and approved by the Institutional Animal Care and Use Committee at the University of Louisville (protocol number IACUC 22221, initial approval on 2 July 2023).

### 2.3. In-Life Monitoring

Animals were observed twice daily for clinical signs starting on day 0 through the end of the study. When it occurs, VEEV-induced encephalitis causes a severe deficit in infected animals in motor activity and morbidity. To evaluate such clinical outcomes, changes in body weight measurement and clinical signs such as activity, grooming, grimace, and neurological behaviors were monitored daily.

### 2.4. Blood Cell Count

The animals were anesthetized under isoflurane, and blood was collected by using the cardiac puncture technique using a 1 mL syringe and 25G needle. Blood was transferred into BD Microtainer tubes containing K2EDTA and mixed well. The samples were then shipped to the Regional Biocontainment Laboratory, and whole blood was processed for a complete blood count using the Hemavet 950 (Drew Scientific, Plantation, FL, USA) within 48 h after blood collection.

### 2.5. Brain Tissue Harvest, Fixation, and Histopathology

After mice were humanely euthanized, total body perfusion was performed to remove the blood from the tissue by manually pushing PBS into the left ventricle via a 23G butterfly needle out to an incision at the right atrium. The brain was then removed and separated into left and right hemispheres. The right hemisphere was placed into an embedding cassette and submerged in 10% buffered formalin for 12–24 h. The embedded tissues were then subjected to paraffin embedding, followed by microtome sectioning. Tissue sectioning, hematoxylin and eosin (H&E) staining, and pathology scoring were all carried out by the Iowa State University Veterinary Medical Pathology. Tissues were evaluated for any pathological findings by a certified pathologist.

### 2.6. Tissue Homogenization

The spinal cord, cerebral cortex, cerebellum, and olfactory bulbs of the left hemisphere were separated and placed into individual homogenization tubes. Additionally, the liver and spleen were collected. The harvested tissues were homogenized in 2 mL tubes prefilled with sterilized 0.5 mm silica beads (Benchmark Scientific Inc. Sayreville, NJ, USA) with two one-minute cycles (Omni BEAD RUPTOR 4) while being kept on ice throughout the entire process. Once homogenized, the tissues were aliquoted into Eppendorf tubes and stored in the –80 °C freezer.

### 2.7. Detection of Virus by Plaque Assay

This study employed two different methods to detect VEEV in the CNS: virus titrations using plaque assay in cell culture and RNAscope assays to detect any replicating and residual viral RNA. Briefly, 6 samples from each group were serially diluted 10-fold in a virus infectious media (VIM: MEM-E, 10% fetal bovine serum, and 25 mM HEPES). Then, 166 µL per well of serially diluted samples were used to infect Vero 76 cells grown overnight in 24-well plates. After one hour of incubation at 37 °C, the supernatant was removed, and the cells were washed with PBS, 0.5 mL per well. Methylcellulose overlay medium (0.7% methylcellulose in VIM) was added to the cell plates and incubated for 3 days in a 37 °C CO_2_ incubator. Virus plaques were developed by using 2% paraformaldehyde and 0.5% crystal violet solution.

### 2.8. Detection of Virus by RNA/Scope Assay

To validate the virus brain titrations and to gain spatial information on virus replication in the brain, the RNAscope assay was implemented. Paraffin-embedded brain tissues from 3 samples per group were sectioned in the sagittal direction, then mounted on glass slides. Viral signal was detected using RNAscope 2.5 HD Detection Reagent—Brown kit (ACD, Cat. # 322310), following the manufacturer’s instructions with a VEEV probes set (ACD, Cat. # 404501). After DAB staining and coverslip mounting, final slides were scanned using a Hamatsu NanoZoomer SQ slide scanner for digital image recording.

### 2.9. Viral Genome Sequence Analysis

Vero 76 cells grown in a 12-well plate overnight (20,000 cells per well) were infected with virus at an MOI of 0.01, and the next day, total RNA was isolated from the infected cells using RNAZol RT (MRC Inc., Cincinnati, OH, USA) following the manufacturer’s protocol. After removing RNA secondary structures by heating to 65 °C for 5 min and placing on ice for another 5 min, cDNA synthesis was performed using 2 µM random hexamers (IDT), RT Buffer, RNaseOUT Ribonuclease Inhibitor, 0.5 mM dNTPs, and Maxima H-minus Reverse Transcriptase (Thermo Fisher Scientific, Waltham, MA, USA ), and the structural and non-structural genes were PCR amplified separately using GC buffer, 0.2 mM dNTPs, Phusion DNA Polymerase (Thermo Scientific), and 0.5µM of each primer (IDT)with a protocol of: 98 °C for 30 s, 30 cycles of 98 °C for 10 s, an annealing temperature of 57.5 °C for 20 s, and 72 °C for 5 min, then 72 °C for 5 min on a S1000 Thermal Cycler (Bio-Rad). Each amplicon was pooled and purified using a 0.5x bead-to-sample volume ratio of KAPA Pure magnetic beads (Roche) and eluted in nuclease-free water. 1 µg of each sample was used in the Nanopore Ligation Sequencing Native Barcode Kit 24 V14. Sequencing was carried out using a MinION Mk1c sequencer with R10.4.1 Flongle flow cells for 20 h with high-accuracy basecalling (Oxford Nanopore Technologies, Oxford, UK). Minimap2 [20] was used to align the fastq files that met the Phred quality score threshold to the reference genome sequence (VEEV GenBank: L01442.2), and mpileup [21] from samtools (now bcftools) was used to identify single-nucleotide polymorphism, including their frequencies at each position in the target region. For the sequenced samples, the average sequencing depth was 2608x, and the average coverage was 84.61%.

### 2.10. Statistical Analysis

The two-way ANOVA and multiple comparison tests were conducted using GraphPad Prism 10 version 10.3.0.

## 3. Results

### 3.1. Study Design

The overall study design aimed to detect potential neuroinvasion after administrations of the test vaccine at peripheral sites, as shown in Table 1. The current IND vaccine, TC-83, is administered at a 1 × 10^5^ PFU dose in humans. To evaluate any potential neuroinvasion, a 20-fold excess dose (2 × 10^6^ PFU per dose per mouse) was used. Inoculation of vaccines is expected to establish a local, limited replication of the vaccine at the injection site. Attenuated strains of VEEV are not expected to have considerable ability to invade the CNS of immunocompetent mice (e.g., BALB/c strain) when they are inoculated via IM or SC route [12]. Each group consisted of 10 animals per time point (DPI2 and 6) with the same number of males and females. Animals were monitored twice daily for body weight changes and clinical scores until the end of the study (i.e., DPI6 see below). DPI 2 and 6 were chosen for neuroinvasive potential assessment because VEEV establishes the CNS infection in mice within 24 h following parenteral inoculation, with lethal outcomes occurring 8–9 days post-infection [22].

### 3.2. V4020 Showed Lower Degree Adverse Effects than Tc-83

To evaluate adverse effects induced by vaccination, several clinical signs were analyzed, including body weight, activity (ACT), grimace (GRI), grooming GRO), and neurological (NEU) abnormality with a scale of 0–4 (none to severe). The mock group injected with PBS showed no changes in body weight over the course of this study. The groups vaccinated with VEEV TC-83 or V4020 via the SC or IM routes did not show any noticeable decrease in body weight compared to the mock group, showing no morbidity in terms of body weight (See Supplementary Figure S2).

**Figure 1:**
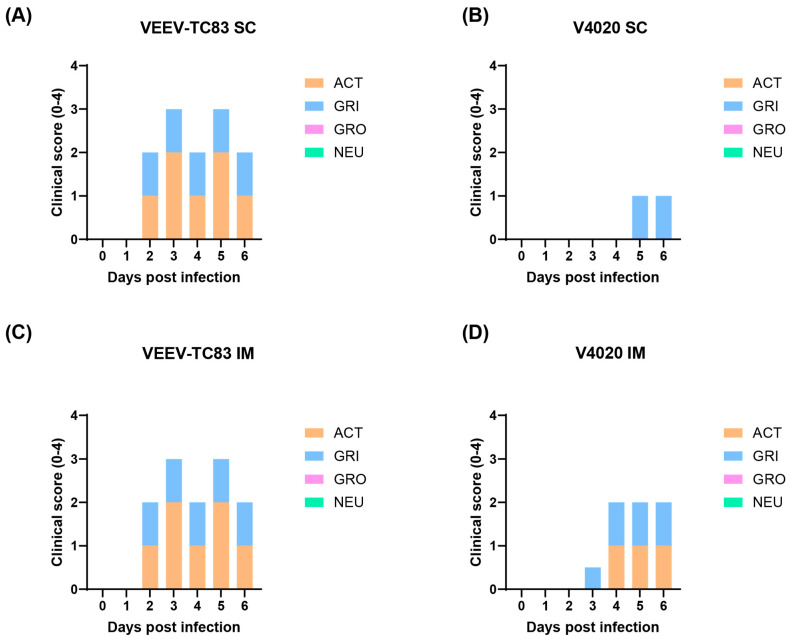
Clinical signs of activity (ACT), grimace (GRI), grooming GRO), and neurological (NEU) abnormality from mice infected with TC-83 and V4020. Clinical signs from mice infected with TC-83 (**A**,**C**) and V4020 (**B**,**D**) after SC (**A**,**B**) and IM (**C**,**D**) administration. The scores on *y*-axis represent the mean scores from 10 mice. Scores were presented with a scale of 0–4 (none to severe) in the stacked column format.

The mock group injected with PBS did not show any clinical signs, and they behaved normally (Figure 1). The TC-83 group vaccinated via the SC route or the IM route demonstrated a moderate level of weakness and slower movements (activity). Animals demonstrated the facial expression of pain and squinting (grimace), starting from 2 days post-infection (DPI 2) until the end of study (DPI 6). In contrast, the V4020 group, either vaccinated via the SC or the IM, demonstrated low-to-moderate level of the clinical signs starting from DPI 3. Some mice from the IM injection group showed a lower activity starting from DPI 4 until the end of the study (DPI 6). In conclusion, compared to VEEV TC-83, in both the SC and IM infection model, the V4020 group clearly showed a lower degree of clinical morbidity signs than that of TC-83 (Figure 1).

### 3.3. V4020 and TC-83 Induced a Low Degree of Changes in the Blood Cell Composition After Administration

**Figure 2:**
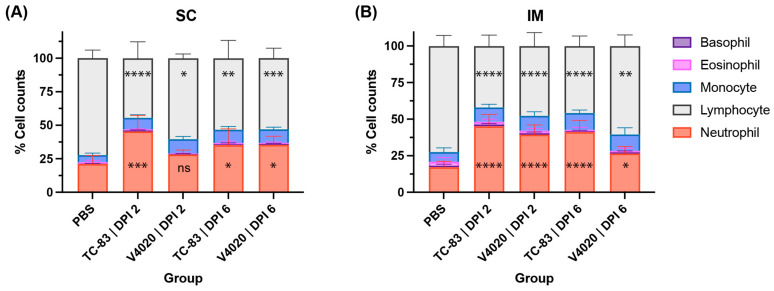
Blood cell type analysis after vaccination with VEEV TC-83 (**A**) and V4020 (**B**). Graphs show mean ± S.D. from 10 samples per group. *, *p* ≤ 0.05; **, *p* ≤ 0.01; ***, *p* ≤ 0.001; ****, *p* ≤ 0.0001 by Dunnett’s multiple comparison test compared to the PBS control.

To evaluate hematological reactions following the vaccination with V4020, total blood samples were subjected to blood cell counting analysis (Figure 2). The mock group harvested at DPI 5 was used as the baseline with a typical blood cell composition of approximately 21% of neutrophils and 72% of lymphocytes. The group vaccinated with VEEV TC-83 via the SC route (Figure 2A) showed a significantly higher proportion of neutrophils (approximately 45%) and a lower proportion of lymphocytes (approximately 44%) at DPI 2. This observation indicates the animals have developed immune responses from the vaccination. The extent of the changes decreased at DPI 6 compared to DPI 2; however, it remained higher than those of the mock group with a higher proportion of neutrophils (approximately 35%) and a lower proportion of lymphocytes (approximately 53%) at DPI 6.

The group vaccinated with V4020 through the SC route showed similar but less pronounced changes, an increase in neutrophils to approximately 28% and a decrease in lymphocytes to approximately 60% at DPI 2 compared to TC-83 vaccinated mice. These changes were maintained at DPI 6, showing a moderate increase in the proportion of neutrophils (approximately 35%) and a decrease in lymphocytes (lymphopenia, approximately 53%) compared to DPI2.

These findings were largely in line with hematology results in the IM vaccinated groups, with slight variations, including the overall higher neutrophil counts. One noticeable difference from the SC study was that neutrophil counts for the V4020 vaccine group were higher at DPI 2 (39%) than DPI6 (27%) for the IM vaccination group (Figure 2B). Overall, after SC or IM administration, both V4020- and TC-83-vaccinated mice demonstrated increases in neutrophil and a decrease in lymphocyte ratio, indicating immunological responses from the vaccination at the blood cell level. The extent of these changes by V4020 was less prominent than those of the TC-83-inoculated group, indicating a lower degree of reactogenicity compared to the TC-83 group.

### 3.4. Detection of Neuroinvasion in TC-83 Infected Mice

To evaluate the sign of neuroinvasion in the animals that received the test vaccines, the brains were harvested at DPI 2 and DPI 6, and the viral loads from four parts of the brain, namely the olfactory bulb, the cerebrum cortex, the spinal cord, and the cerebellum were enumerated (6 animals, mixed sex, from each group). The mock-infected control group did not show any infectious virus in the CNS. For both TC-83 and V4020 infected groups, no infectious viruses were detected in the CNS at DPI 2. However, some animals that received TC-83 showed a detectable amount of virus in the CNS at DPI 6. Among the SC administration group, 50% (three out of six) brain samples showed infectious virus, two in the cerebral cortex and one in the spinal cord (Table 2). For the IM vaccination group (Table 3), two out of six samples showed a detectable amount of virus in the brain. However, none of the brain samples from the V4020 administered mice showed any detectable level of virus, which highlights the lower risk of V4020 compared to TC-83.

**Figure 3:**
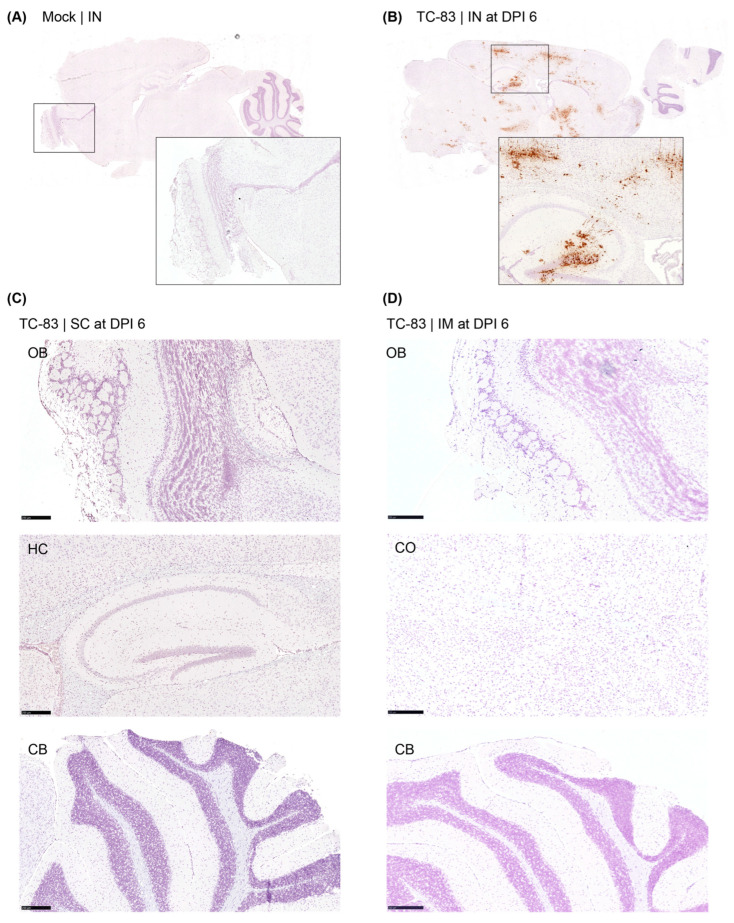
Detection of viral RNA in the brain tissues by RNAScope analysis from TC-83-vaccinated mouse. Brain tissues from mock (**A**), TC-83 IN (**B**), TC-83 SC (**C**), and TC-83 IM (**D**) administrations were subjected to RNAScope assay with a probe specific to VEEV (brown stain). OB, olfactory bulbs; CO, cerebral cortex; H.C., hippocampus; CB, cerebellum. Representative images from three samples per group. Scale bars represent 250 µm.

**Figure 4:**
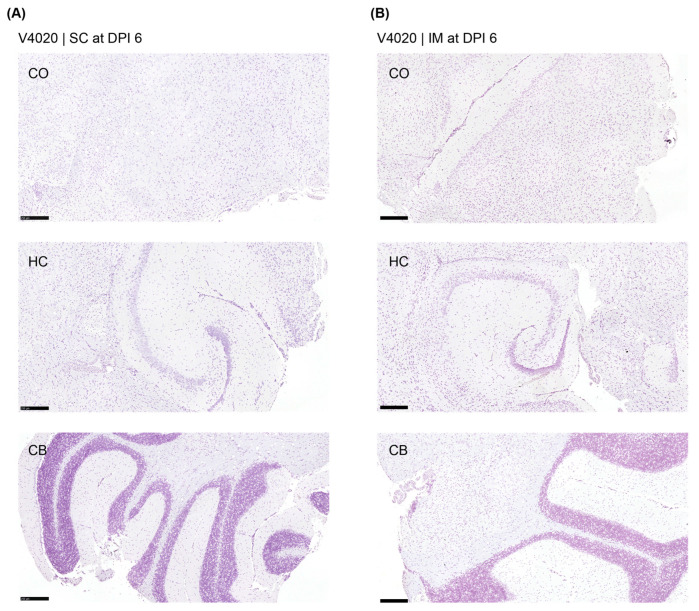
Detection of viral RNA in the brain tissues by RNAScope analysis from V4020-vaccinated mice. Brain tissues from V4020 SC (**A**) and V4020 IM (**B**) administrations were subjected to RNAScope assay with a probe specific to VEEV (brown stain). CO, cerebral cortex; H.C., hippocampus; CB, cerebellum. Representative images from three samples. Scale bars represent 250 µm.

We employed the RNAScope assay to detect infectious, replicating virus with respect to location within the brain (three samples per group). As a positive control, brains from mice (strain C3H/Ne) intranasally infected with TC-83 were used (Figure 3). The mock-treated group did not show any VEEV-positive signal anywhere in the brain tissues, and the positive control samples showed a strong positive signal for the virus, as expected. However, no VEEV-specific signal was detected from the brains of the V4020 or the TC-83-vaccinated group (Figure 4).

### 3.5. Pseudoreversion of the Neuroinvasive Tc-83 Population

To understand what changes in the viral genome could be associated with the neuroinvasion of TC-83, we sequenced the genome of the TC-83 virus isolated from the brains. Total cellular RNAs from Vero 76 cells infected with the samples were subjected to PCR-amplicon based sequencing (Table 4). We found that all three isolates had two non-synonymous mutations: (1) E2 R120I, and (2) E1 V80A. The E2 R120I mutation was caused by a single mutation at nucleotide position 8922 G/T, of which the codon is responsible for the attenuation mutation of TC-83 (Arg120) compared to its processor, Trinadad Donkey strain (TrD, Thr120). Sequencing analysis of the inoculum samples of both TC-83 and V4020 did not detect these mutations (data not shown), indicating the mutation might be selected during replication within the animals.

### 3.6. TC-83 and V4020 Did Not Induce Pathological Changes in the Brains and Other Organs

**Figure 5:**
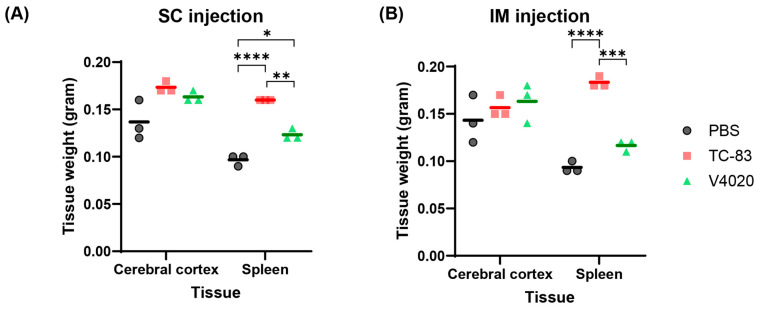
Tissue weight analysis of TC-83 and V4020 vaccinated mice. Tissue weight analysis of SC (**A**) or IM (**B**) infection of VEEV TC83 and V4020 in the Balb/c mouse (*n* = 3) model at DPI 6. *, *p* ≤ 0.05; **, *p* ≤ 0.01; ***, *p* ≤ 0.001; ****, *p* ≤ 0.0001 by Tukey’s multiple comparison test.

To evaluate if the vaccines could cause any pathogenic effect at the gross level, major organs were tested for macroscopic gross pathology evaluation. No macroscopic pathology was noticed in the brain or in the abdominal cavity in all the animals, and no signs of irritation (swelling, redness, etc.) were seen at the injection sites for both SC and IM injection groups (Table 5). Clinical observation did not reveal any apparent abnormalities or changes in the size or appearance of major organs. Weight analysis of the brain (cerebral cortex) and spleen showed that the brain weight did not show any significant difference in any of the test groups compared to the mock group (Figure 5). However, spleens from the TC83 and V4020 were larger than those from the mock group, with statistical significance between groups (Tukey’s multiple comparison test), although small group size (*n* = 3) has limitations for statistical analysis. Interestingly, spleens from the TC-83 IM vaccinated group showed a higher weight compared to those from V4020, potentially indicating higher immunogenic activity by TC-83 than V4020.

To address concerns about potential neuroinvasion of the vaccine, tissue histopathology of the brains was conducted. As a positive control, brains from mice intranasally infected with TC-83 were used. The intranasally infected TC-83 group showed a moderate degree of lesions in the meninges and cerebrum in all samples (*n* = 10). For both TC-83 and V4020-vaccinated groups, however, no microscopic lesions were detected in any of the brain samples from either SC or IM administration route groups (*n* = 6) at either DPI 2 or DPI 6. In conclusion, the legacy VEEV vaccine, VEEV TC-83, and the V4020 did not induce any tissue pathology in the brain after SC or IM injection, which are the routes of administration for humans.

## 4. Discussion

Although the minimum level of neuroinvasion may not necessarily indicate the safety of the vaccine, understanding of the potential of neuroinvasion is critical information to evaluate the safety of live-attenuated vaccines for neurotropic viruses such as VEEV. Previously, we have reported that in a murine model, VEEV V4020 had improved safety and genetic stability in comparison with the parental TC-83 vaccine [23]. We extended our study to compare the safety profiles of V4020 and TC-83 in this study. Particularly, we evaluated the neuroinvasion potential of V4020, a TC-83-based VEEV vaccine with a rationally designed strategy for attenuation as well as for prevention of potential reversion. The immunogenicity and V4020 vaccine-induced protection from VEEV viremia after challenge have been previously demonstrated in a non-human primate model [14]. Here, we employed immunocompetent mice with a vaccine dose of 2 × 10^6^ PFU, a 20-fold higher than the expected 10^5^ PFU vaccination dose, either SC or IM, the intended routes of administration for the V4020 vaccine. We compared the neuroinvasion potential and any adverse effects to TC-83, the legacy VEEV vaccine strain, as its comparator. Overall, our study found that adverse effects from V4020 vaccination were significantly lower compared to the comparator VEEV TC-83, based on the clinical scores and blood cell counts. However, neither vaccine seems to induce changes in hematology at a pathological level, considering the normal mouse blood cell composition. In addition, our results showing the minimal or lack of neurotropism from both TC-83 and V4020 are largely consistent with other reports [12,22]. Also, these results demonstrated a clear difference between these vaccines from the parental strain of VEEV TrD in neuroinvasive potential, which induces a lethal neurotropic infection within 8–9 days after a subcutaneous injection, resulting in viral loads in the brain of 10 ^8−9^ pfu/gram [12,24].

Importantly, we found that live TC-83 vaccine virus can establish a CNS-infection at a rather significant rate (30–50% frequency) after SC or IM administration in mice. While neuroinvasion of virulent VEEV after peripheral route infection is well documented, to the best of our knowledge, no previous studies have shown neuroinvasion of TC-83 following peripheral infection in adult mice other than intranasal infection. While our finding could be due to the high amount of virus inoculum (2 × 10^6^ PFU per dose per mouse, 20-fold higher than the expected vaccine dose), this observation of the neuroinvasion by TC-83 vaccine is in line with the clinical observations with the TC-83 vaccine in humans, as it was used as part of Special Immunization Programs to vaccinate medical workers at risk of VEEV infection. Up to 20% of TC-83 vaccine recipients experienced adverse reactions, including mild to medium neurological symptoms [8,9,10].

**Figure 6:**
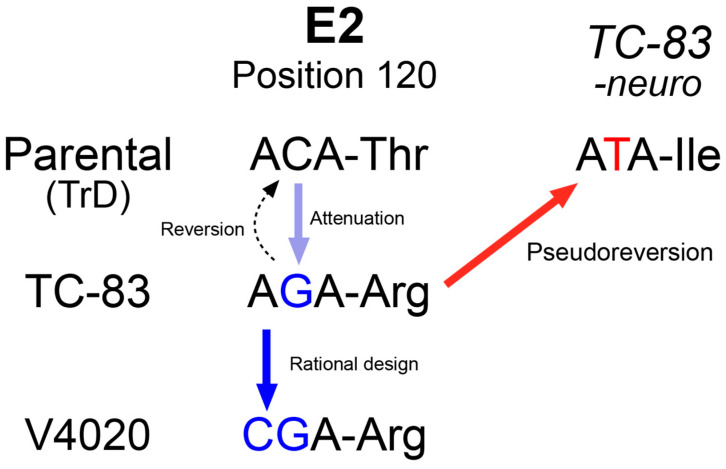
Pseudoreversion of TC-83 and the strategy implemented in V4020 to minimize the risk.

TC-83 virus recovered from the CNS (i.e., neurotropic TC-83) harbored two unique mutations in the E2 and E1 genes, which play critical roles in virus–receptor interaction. The mutations, particularly the E2 R120I mutation, that we found from the TC-83 virus recovered from the CNS (i.e., neurotropic TC-83) were of special interest. The R120 residue is well known to confer attenuation compared to its virulent parental strain, TrD, which encodes Thr [25]. The T120R mutation in TC-83, a change from a neutral to a positive charge amino acid, is likely to promote the binding with heparin sulfate in cell cultures and to decrease with its putative receptor in vivo [26,27,28]. Interestingly, the neurotropic TC-83 population encoded the R120I mutation, which is not a true reversion as the Arg residue has changed to Ile, not Thr. We believe that this change is a pseudoreversion, restoring the phenotype lost by changing the residue to a neutral amino acid (Figure 6). However, we did not validate that this pseudoreversion is the key factor to regain the neuroinvasion activity of TC-83 due to the dual-use concern, because such an introduction to TC-83 might be a gain-of-function for an existing vaccine.

In contrast, the lack of detectable neuroinvasion by V4020 suggests a higher safety profile of V4020 in regard to neuroinvasion potential. While we cannot directly conclude, this lack of neuroinvasion of V4020 is presumably due to the fact that the codon for E2-R120 was engineered to the CGA codon, which requires a double mutation for the first two positions of the codon for reversion. A previous study analyzing virus population sequence V4020 RNA showed that no SNPs of 1% or higher frequency were detected at each nucleotide of the codon, suggesting a low probability of a reversion or a pseudoreversion.

Alternatively, this lack of neuroinvasion could be because of the lower V4020 viremia titer in the circulation for the higher level of attenuation of V4020, as compared to the TC-83 virus, as higher viremia could increase the chance of neuroinvasion from circulation. After SC inoculation, virulent VEEV reaches viremia 10^5^–10^7^ PFU/mL in the serum, while viremia for attenuated variants varies from undetectable to rarely detectable depending on the attenuating mutation [12]. Attenuating rearrangement of the capsid and glycoproteins in V4020 would have contributed to the decrease, if any, in viral load in the blood.

Although we detected a virus in the brain following the vaccination with TC-83, we did not find pathological outcomes nor RNA detection by RNAScope assay at the histopathology level. This lack of consistency could be due to the nature of the approach, utilizing a small section of the tissue, rather than the entire brain, for virus isolation. Another explanation is the premature development of neuropathology, as the neuroinvasion could have happened at a later time point. Another important consideration is the vaccine virus population diversity, as was shown for other live-attenuated vaccines. For example, the yellow fever virus (YFV) vaccine YF-17D is safely used worldwide to prevent infections with YFV. However, a small percentage, 0.000012–0.00002% of vaccinated patients can develop post-vaccination neurological syndrome. Studies of YF-French neurotropic vaccine (YF-FNV) and YF-17D variant have identified several neuroinvasive genetic variants in the vaccine viruses [29,30]. In the study, YF-17D had fewer genetic variations than those of YF-FNV, and it was concluded that viral population diversity is a critical factor for YFV vaccine neuroinvasiveness [30]. Our NGS studies to compare experimental Chikungunya virus (CHIKV) vaccines revealed that the vaccine virus derived from an infectious clone has fewer variations at attenuating sites, when compared to the classic CHIKV 181/25 vaccine derived by multiple passages [31]. Likewise, compared to the TC-83 vaccine virus, which has been generated by multiple passages in tissue culture, V4020 was derived from an iDNA infectious clone, which might have mitigated the genetic variants as compared to the TC-83 vaccine [14,15]. Detailed studies on the relationship between the genetic variation and the effect on neuroinvasiveness for V4020 and the recently approved CHIKV vaccine [32] are currently planned.

## 5. Conclusions

In conclusion, while the legacy VEEV vaccine TC-83 poses neuroinvasion potential via a pseudoreversion, our study establishes the experimental V4020 vaccine as a safer alternative vaccine candidate with advantageous safety features, including reduced or no neuroinvasion risk.

## Figures and Tables

**Table 1:** Study design for neuroinvasion of TC-83 and V4020 in an immune-competent mouse model.

	Goal/Purpose	Mouse Strain	Inoculum	Infection Route	Sacrifice Day (DPI)	No. of Animals
Group 1	Negative control	Balb/C	PBS	IM	5	5 males and 5 females
Group 2	IM route, comparator	Balb/C	TC-83, 2 × 10^6^ PFU/ms	IM	2	5 males and 5 females
					6	5 males and 5 females
Group 3	IM route, test article	Balb/C	V4020, 2 × 10^6^ PFU/ms	IM	2	5 males and 5 females
					6	5 males and 5 females
Group 4	SC route, comparator	Balb/C	TC-83, 2 × 10^6^ PFU/ms	SC	2	5 males and 5 females
					6	5 males and 5 females
Group 5	SC route, test article	Balb/C	V4020, 2 × 10^6^ PFU/ms	SC	2	5 males and 5 females
					6	5 males and 5 females

**Table 2:** Virus titers in the CNS samples after the SC route vaccine administration.

Inoculum	Animal ID	Sex	DPI 6			
			OB	CO	CB	SP
TC-83	1	Female	N.D.	2.3 × 10^6^	1.6 × 10^3^	N.D.
	2	Female	N.D.	2.2 × 10^3^	N.D.	N.D.
	3	Female	N.D.	N.D.	N.D.	N.D.
	4	Male	N.D.	N.D.	N.D.	N.D.
	5	Male	N.D.	N.D.	N.D.	7.5 × 10^2^
	6	Male	N.D.	N.D.	N.D.	N.D.
V4020	1	Female	N.D.	N.D.	N.D.	N.D.
	2	Female	N.D.	N.D.	N.D.	N.D.
	3	Female	N.D.	N.D.	N.D.	N.D.
	4	Male	N.D.	N.D.	N.D.	N.D.
	5	Male	N.D.	N.D.	N.D.	N.D.
	6	Male	N.D.	N.D.	N.D.	N.D.

Viral load (pfu/gram); N.D., not detected. OB, olfactory bulbs; CO, cerebral cortex; CB, cerebellum; SP, spinal cord. The limit of detection was 300 pfu/mL sample.

**Table 3:** Virus titers in the CNS samples after the IM route vaccine administration.

Inoculum	Animal ID	Sex	DPI 6			
			OB	CO	CB	SP
TC-83	1	Female	N.D.	N.D.	N.D.	N.D.
	2	Female	N.D.	N.D.	N.D.	N.D.
	3	Female	N.D.	N.D.	N.D.	9.0 × 10^2^
	4	Male	N.D.	N.D.	N.D.	N.D.
	5	Male	N.D.	N.D.	N.D.	1.5 × 10^3^
	6	Male	N.D.	N.D.	N.D.	N.D.
V4020	1	Female	N.D.	N.D.	N.D.	N.D.
	2	Female	N.D.	N.D.	N.D.	N.D.
	3	Female	N.D.	N.D.	N.D.	N.D.
	4	Male	N.D.	N.D.	N.D.	N.D.
	5	Male	N.D.	N.D.	N.D.	N.D.
	6	Male	N.D.	N.D.	N.D.	N.D.

Viral load (pfu/gram); N.D., not detected. OB, olfactory bulbs; CO, cerebral cortex; CB, cerebellum; SP, spinal cord. The limit of detection was 300 pfu/mL sample.

**Table 4:** Sequence analysis of TC-83 viruses isolated from CNS of mice.

Gene/a.a, Position	TrD Reference	TC-83 Inoculum	V4020 Inoculum	Isolate 1 */Animal 1 CB	Isolate 2 */Animal 1 CO	Isolate 3 */Animal 2 CO
E2/120	ACA/Thr	AGA/Arg	CGA/Arg	ATA/Ile	ATA/Ile	ATA/Ile
E1/80	GTC/Val	GTC/Val	GTC/Val	GCC/Ala	GCC/Ala	GCC/Ala

* All samples were from TC-83-infected brains, harvested at DPI6. Sequences are shown as codon/amino acid. CO, cerebral cortex; CB, cerebellum.

**Table 5:** A summary of the brain pathology after VEEV vaccinations.

Injection route	Inoculum	DPI	Pathological Findings
IN	PBS	5	No microscopic lesions in the brains (*n* = 10).
	TC-83	6	Minimal to moderate lesions in the meninges and cerebrum in all brains (*n* = 10).
SC	PBS	6	No microscopic lesions in the brain (*n* = 6).
	TC-83	2	No microscopic lesions in the brain (*n* = 6).
		6	No microscopic lesions in the brain (*n* = 6).
	V4020	2	No microscopic lesions in the brain (*n* = 6).
		6	No microscopic lesions in the brain (*n* = 6).
IM	PBS	5	No microscopic lesions in the brain (*n* = 6).
	TC-83	2	No microscopic lesions in the brain (*n* = 6).
		6	No microscopic lesions in the brain (*n* = 6).
	V4020	2	No microscopic lesions in the brain (*n* = 6).
		6	No microscopic lesions in the brain (*n* = 6).

## Data Availability

Data available upon request.

## Supplementary Materials

The following supporting information can be downloaded at: https://www.mdpi.com/article/10.3390/v17081136/s1, Figure S1: Genomic structure of TC-83 (middle) and V4020 (bottom) compared to the wildtype strain, TrD (top); Figure S2: Change of the body weight after the SC (A) or IM (B) vaccination of the test vaccines. % Body weight change compared to DPI 0. n.s., no significant by Two-Way RM ANOVA test or by Sidak multiple comparison (top of the lines); Spplementary Table S1: Sequences of primers used for the study.

## Abbreviations

The following abbreviations are used in this manuscript:

VEEV	Venezuelan equine encephalitis virus
CNS	central nervous system
IM	intramuscular
SC	subcutaneous
IN	intranasal
