# Supplementary material for: V4020 Venezuelan Equine Encephalitis Vaccine: Mitigating Neuroinvasion and Reversion Through Rational Design
Source: Viruses. 2025 Aug 19;17(8):1136. doi: 10.3390/v17081136 (PMC12390694; doi:10.3390/v17081136)
Supplement: Supplementary file 1 [file viruses-17-01136-s001.zip › viruses-3752998-supplementary.pdf]

Supplementary Figure.

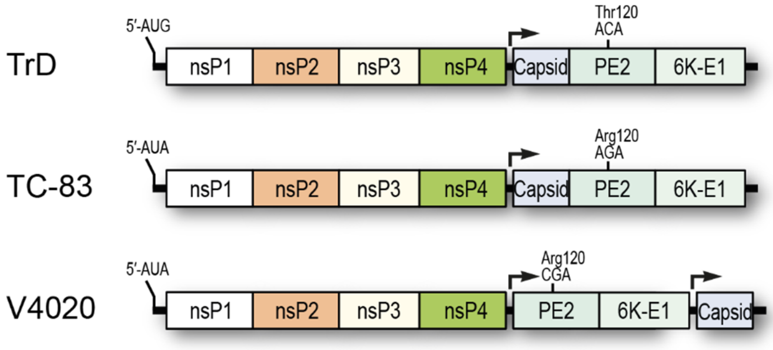

Supplementary Figure S1. Genomic structure of TC-83 (middle) and V4020 (bottom) compared to the wildtype strain, TrD (top).

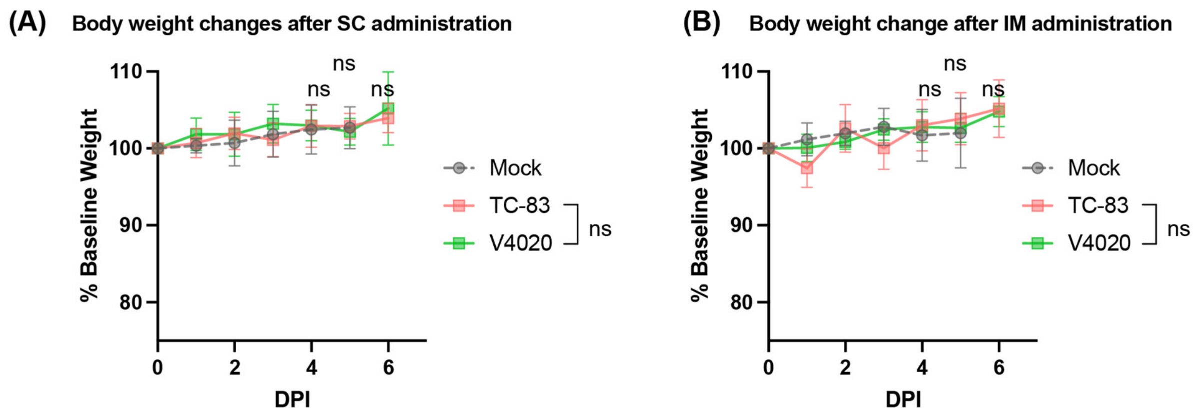

Supplementary Figure S2. Change of the body weight after the SC (A) or IM (B) vaccination of the test vaccines. % Body weight change compared to DPI 0. n.s., no significant by Two-Way RM ANOVA test or by Sidak multiple comparison (top of the lines).

Spplementary Table

Spplementary Table S1. Sequences of primers used for the study

Primer name	Sequences
Forward Structural Primer	5′- GCCCAGACCAATTACCTACCCAAA -3′
Reverse Structural Primer	5′- AGACTATGTCGTAGTCCATTCAGG -3′
Forward Non-structural Primer	5′- ATGGACTACGACATAGTCTAGTCCG -3′
Reverse Non-structural Primer	5′- AATCGCCGCGAGTTCTATGTAA -3′
